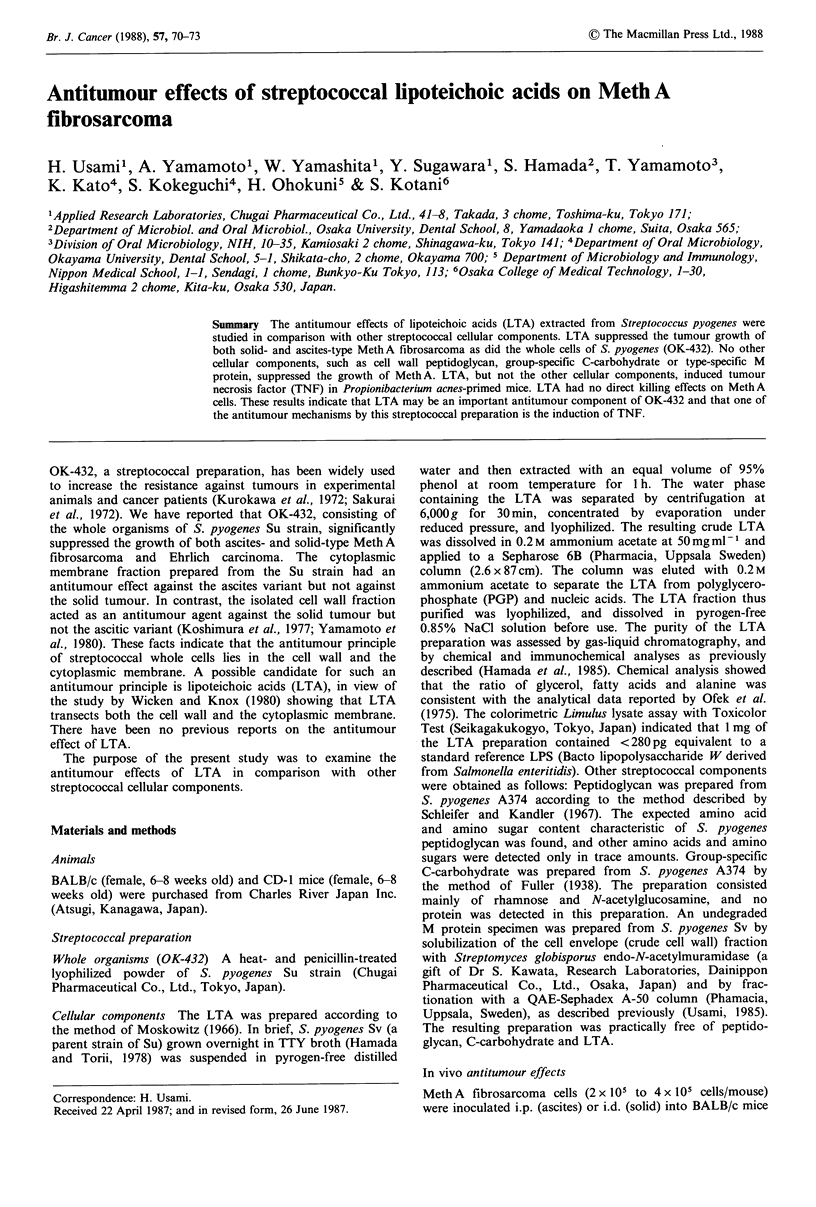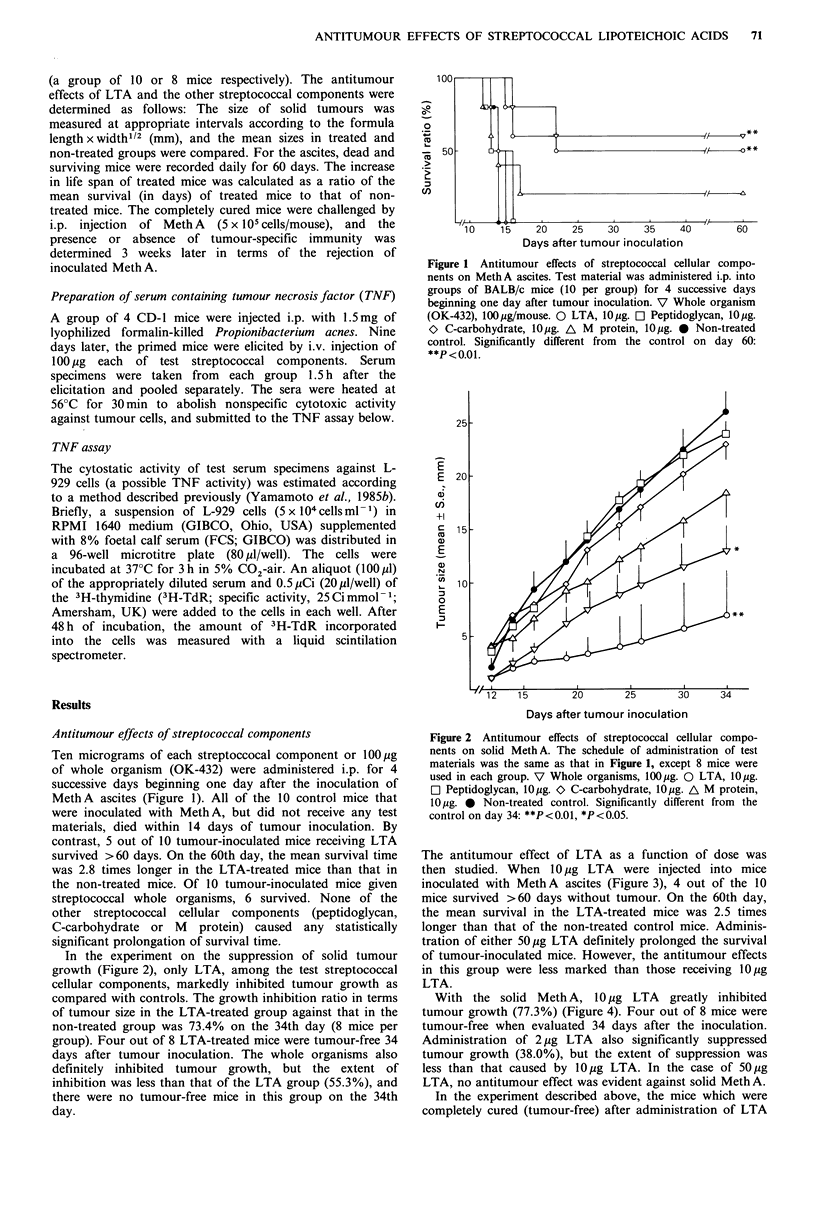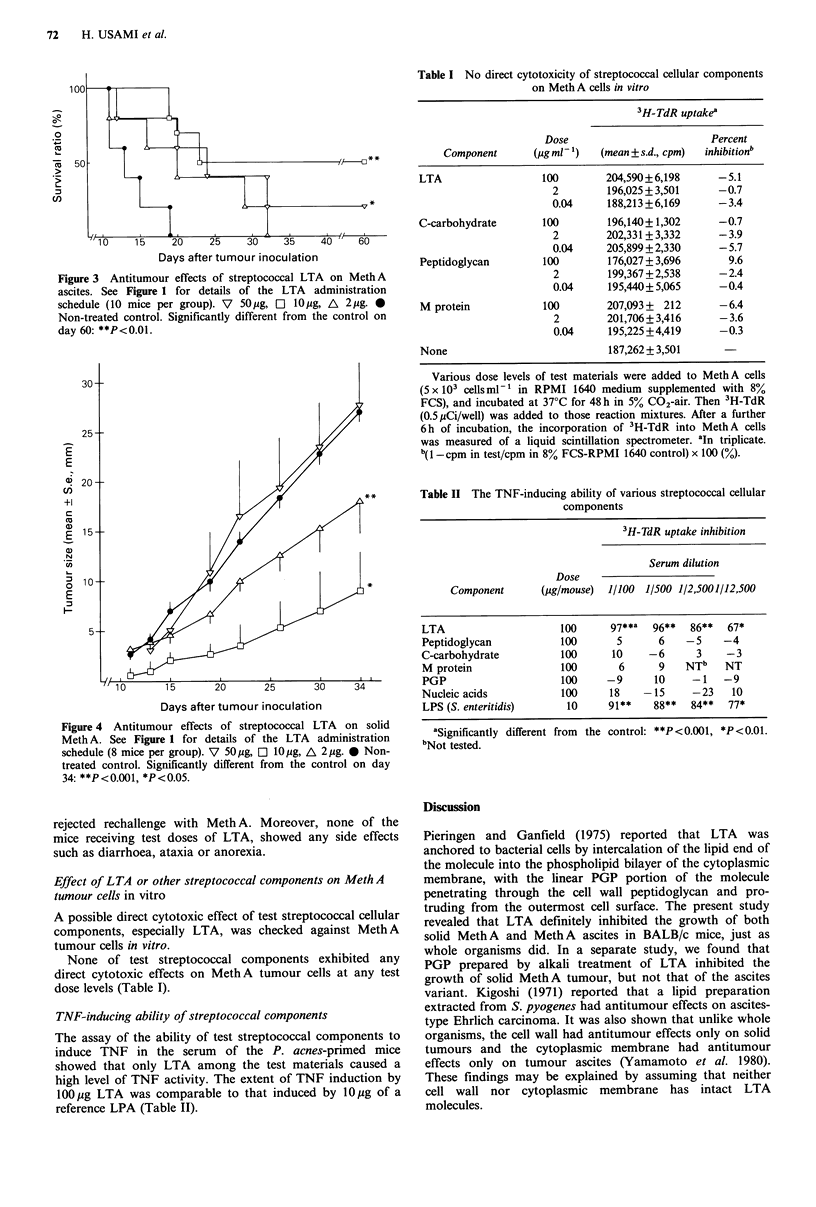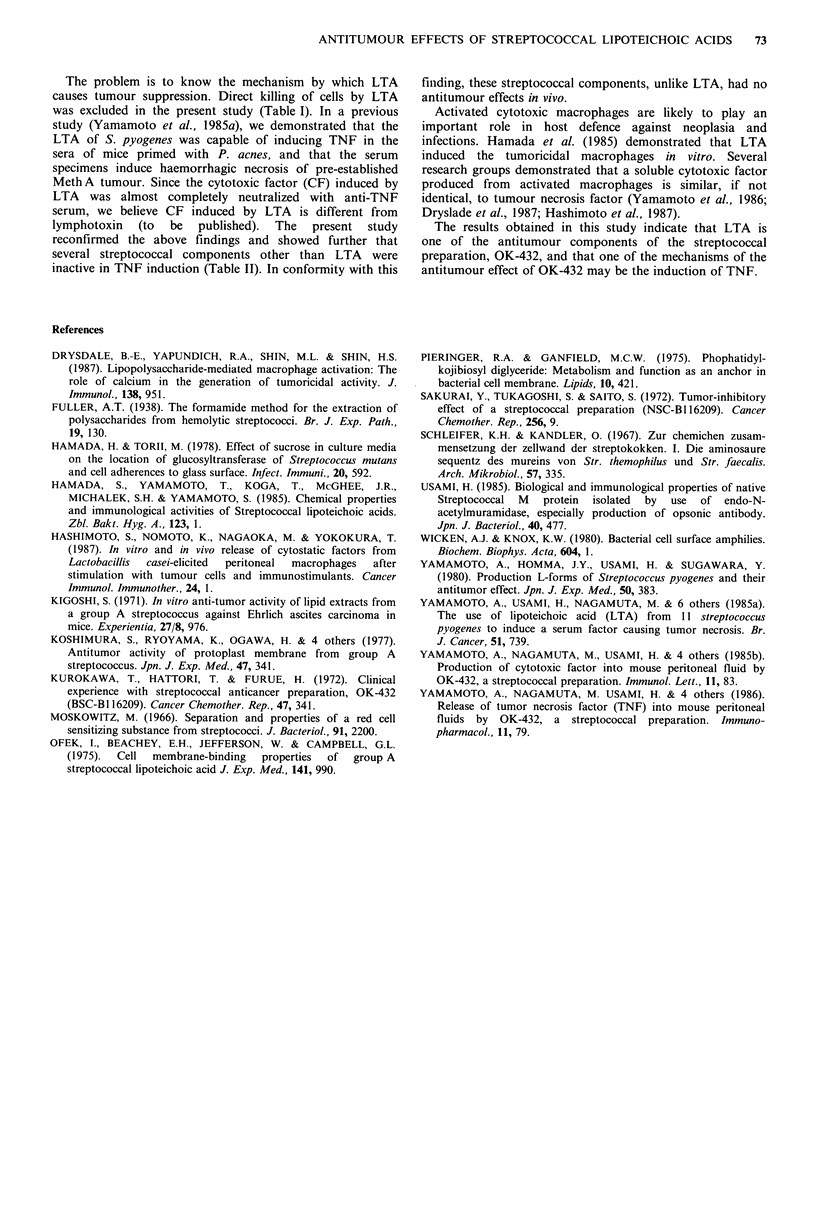# Antitumour effects of streptococcal lipoteichoic acids on Meth A fibrosarcoma.

**DOI:** 10.1038/bjc.1988.11

**Published:** 1988-01

**Authors:** H. Usami, A. Yamamoto, W. Yamashita, Y. Sugawara, S. Hamada, T. Yamamoto, K. Kato, S. Kokeguchi, H. Ohokuni, S. Kotani

**Affiliations:** Applied Research Laboratories, Chugai Pharmaceutical Co., Ltd., Tokyo, Japan.

## Abstract

The antitumour effects of lipoteichoic acids (LTA) extracted from Streptococcus pyogenes were studied in comparison with other streptococcal cellular components. LTA suppressed the tumour growth of both solid- and ascites-type Meth A fibrosarcoma as did the whole cells of S. pyogenes (OK-432). No other cellular components, such as cell wall peptidoglycan, group-specific C-carbohydrate or type-specific M protein, suppressed the growth of Meth A. LTA, but not the other cellular components, induced tumour necrosis factor (TNF) in Propionibacterium acnes-primed mice. LTA had no direct killing effects on Meth A cells. These results indicate that LTA may be an important antitumour component of OK-432 and that one of the antitumour mechanisms by this streptococcal preparation is the induction of TNF.


Br. J. Cancer (1988), 57, 70–73 © The Macmillan Press Ltd., 1988

### Antitumour effects of streptococcal lipoteichoic acids on Meth A fibrosarcoma

H. Usami[1], A. Yamamoto[1], W. Yamashita[1], Y. Sugawara[1], S. Hamada[2], T. Yamamoto[3], K. Kato[4], S. Kokeguchi[4], H. Ohokuni[5] & S. Kotani[6]

[1]*Applied Research Laboratories, Chugai Pharmaceutical Co., Ltd., 41–8, Takada, 3 chome, Toshima-ku, Tokyo 171;* [2]*Department of Microbiol. and Oral Microbiol., Osaka University, Dental School, 8, Yamadaoka 1 chome, Suita, Osaka 565;* [3]*Division of Oral Microbiology, NIH, 10–35, Kamiosaki 2 chome, Shinagawa-ku, Tokyo 141;* [4]*Department of Oral Microbiology, Okayama University, Dental School, 5–1, Shikata-cho, 2 chome, Okayama 700;* [5] *Department of Microbiology and Immunology, Nippon Medical School, 1–1, Sendagi, 1 chome, Bunkyo-Ku Tokyo, 113;* [6]*Osaka College of Medical Technology, 1–30, Higashitemma 2 chome, Kita-ku, Osaka 530, Japan.*

**Summary** The antitumour effects of lipoteichoic acids (LTA) extracted from *Streptococcus pyogenes* were studied in comparison with other streptococcal cellular components. LTA suppressed the tumour growth of both solid- and ascites-type Meth A fibrosarcoma as did the whole cells of *S. pyogenes* (OK-432). No other cellular components, such as cell wall peptidoglycan, group-specific C-carbohydrate or type-specific M protein, suppressed the growth of Meth A. LTA, but not the other cellular components, induced tumour necrosis factor (TNF) in *Propionibacterium acnes*-primed mice. LTA had no direct killing effects on Meth A cells. These results indicate that LTA may be an important antitumour component of OK-432 and that one of the antitumour mechanisms by this streptococcal preparation is the induction of TNF.

OK-432, a streptococcal preparation, has been widely used to increase the resistance against tumours in experimental animals and cancer patients (Kurokawa *et al.*, 1972; Sakurai *et al.*, 1972). We have reported that OK-432, consisting of the whole organisms of *S. pyogenes* Su strain, significantly suppressed the growth of both ascites- and solid-type Meth A fibrosarcoma and Ehrlich carcinoma. The cytoplasmic membrane fraction prepared from the Su strain had an antitumour effect against the ascites variant but not against the solid tumour. In contrast, the isolated cell wall fraction acted as an antitumour agent against the solid tumour but not the ascitic variant (Koshimura *et al.*, 1977; Yamamoto *et al.*, 1980). These facts indicate that the antitumour principle of streptococcal whole cells lies in the cell wall and the cytoplasmic membrane. A possible candidate for such an antitumour principle is lipoteichoic acids (LTA), in view of the study by Wicken and Knox (1980) showing that LTA transects both the cell wall and the cytoplasmic membrane. There have been no previous reports on the antitumour effect of LTA.

The purpose of the present study was to examine the antitumour effects of LTA in comparison with other streptococcal cellular components.

### Materials and methods

### *Animals*

BALB/c (female, 6–8 weeks old) and CD-1 mice (female, 6–8 weeks old) were purchased from Charles River Japan Inc. (Atsugi, Kanagawa, Japan).

### *Streptococcal preparation*

*Whole organisms (OK-432)* A heat- and penicillin-treated lyophilized powder of *S. pyogenes* Su strain (Chugai Pharmaceutical Co., Ltd., Tokyo, Japan).

*Cellular components* The LTA was prepared according to the method of Moskowitz (1966). In brief, *S. pyogenes* Sv (a parent strain of Su) grown overnight in TTY broth (Hamada and Torii, 1978) was suspended in pyrogen-free distilled water and then extracted with an equal volume of 95% phenol at room temperature for 1 h. The water phase containing the LTA was separated by centrifugation at 6,000 $g$ for 30 min, concentrated by evaporation under reduced pressure, and lyophilized. The resulting crude LTA was dissolved in 0.2 M ammonium acetate at 50 mg ml$^{-1}$ and applied to a Sepharose 6B (Pharmacia, Uppsala Sweden) column (2.6 × 87 cm). The column was eluted with 0.2 M ammonium acetate to separate the LTA from polyglycerophosphate (PGP) and nucleic acids. The LTA fraction thus purified was lyophilized, and dissolved in pyrogen-free 0.85% NaCl solution before use. The purity of the LTA preparation was assessed by gas-liquid chromatography, and by chemical and immunochemical analyses as previously described (Hamada *et al.*, 1985). Chemical analysis showed that the ratio of glycerol, fatty acids and alanine was consistent with the analytical data reported by Ofek *et al.* (1975). The colorimetric *Limulus* lysate assay with Toxicolor Test (Seikagakukogyo, Tokyo, Japan) indicated that 1 mg of the LTA preparation contained <280 pg equivalent to a standard reference LPS (Bacto lipopolysaccharide *W* derived from *Salmonella enteritidis*). Other streptococcal components were obtained as follows: Peptidoglycan was prepared from *S. pyogenes* A374 according to the method described by Schleifer and Kandler (1967). The expected amino acid and amino sugar content characteristic of *S. pyogenes* peptidoglycan was found, and other amino acids and amino sugars were detected only in trace amounts. Group-specific C-carbohydrate was prepared from *S. pyogenes* A374 by the method of Fuller (1938). The preparation consisted mainly of rhamnose and *N*-acetylglucosamine, and no protein was detected in this preparation. An undegraded M protein specimen was prepared from *S. pyogenes* Sv by solubilization of the cell envelope (crude cell wall) fraction with *Streptomyces globisporus* endo-*N*-acetylmuramidase (a gift of Dr S. Kawata, Research Laboratories, Dainippon Pharmaceutical Co., Ltd., Osaka, Japan) and by fractionation with a QAE-Sephadex A-50 column (Phamacia, Uppsala, Sweden), as described previously (Usami, 1985). The resulting preparation was practically free of peptidoglycan, C-carbohydrate and LTA.

### In vivo *antitumour effects*

Meth A fibrosarcoma cells ($2\times10^5$ to $4\times10^5$ cells/mouse) were inoculated i.p. (ascites) or i.d. (solid) into BALB/c mice

Correspondence: H. Usami.
Received 22 April 1987; and in revised form, 26 June 1987.

(a group of 10 or 8 mice respectively). The antitumour effects of LTA and the other streptococcal components were determined as follows: The size of solid tumours was measured at appropriate intervals according to the formula length × width$^{1/2}$ (mm), and the mean sizes in treated and non-treated groups were compared. For the ascites, dead and surviving mice were recorded daily for 60 days. The increase in life span of treated mice was calculated as a ratio of the mean survival (in days) of treated mice to that of non-treated mice. The completely cured mice were challenged by i.p. injection of Meth A ($5 \times 10^5$ cells/mouse), and the presence or absence of tumour-specific immunity was determined 3 weeks later in terms of the rejection of inoculated Meth A.

*Preparation of serum containing tumour necrosis factor (TNF)*

A group of 4 CD-1 mice were injected i.p. with 1.5 mg of lyophilized formalin-killed *Propionibacterium acnes*. Nine days later, the primed mice were elicited by i.v. injection of 100 µg each of test streptococcal components. Serum specimens were taken from each group 1.5 h after the elicitation and pooled separately. The sera were heated at 56°C for 30 min to abolish nonspecific cytotoxic activity against tumour cells, and submitted to the TNF assay below.

*TNF assay*

The cytostatic activity of test serum specimens against L-929 cells (a possible TNF activity) was estimated according to a method described previously (Yamamoto *et al.*, 1985*b*). Briefly, a suspension of L-929 cells ($5 \times 10^4$ cells ml$^{-1}$) in RPMI 1640 medium (GIBCO, Ohio, USA) supplemented with 8% foetal calf serum (FCS; GIBCO) was distributed in a 96-well microtitre plate (80 µl/well). The cells were incubated at 37°C for 3 h in 5% $CO_2$-air. An aliquot (100 µl) of the appropriately diluted serum and 0.5 µCi (20 µl/well) of the $^3$H-thymidine ($^3$H-TdR; specific activity, 25 Ci mmol$^{-1}$; Amersham, UK) were added to the cells in each well. After 48 h of incubation, the amount of $^3$H-TdR incorporated into the cells was measured with a liquid scintilation spectrometer.

### Results

*Antitumour effects of streptococcal components*

Ten micrograms of each streptoccocal component or 100 µg of whole organism (OK-432) were administered i.p. for 4 successive days beginning one day after the inoculation of Meth A ascites (Figure 1). All of the 10 control mice that were inoculated with Meth A, but did not receive any test materials, died within 14 days of tumour inoculation. By contrast, 5 out of 10 tumour-inoculated mice receiving LTA survived >60 days. On the 60th day, the mean survival time was 2.8 times longer in the LTA-treated mice than that in the non-treated mice. Of 10 tumour-inoculated mice given streptococcal whole organisms, 6 survived. None of the other streptococcal cellular components (peptidoglycan, C-carbohydrate or M protein) caused any statistically significant prolongation of survival time.

In the experiment on the suppression of solid tumour growth (Figure 2), only LTA, among the test streptococcal cellular components, markedly inhibited tumour growth as compared with controls. The growth inhibition ratio in terms of tumour size in the LTA-treated group against that in the non-treated group was 73.4% on the 34th day (8 mice per group). Four out of 8 LTA-treated mice were tumour-free 34 days after tumour inoculation. The whole organisms also definitely inhibited tumour growth, but the extent of inhibition was less than that of the LTA group (55.3%), and there were no tumour-free mice in this group on the 34th day.

**Figure 1** Antitumour effects of streptococcal cellular components on Meth A ascites. Test material was administered i.p. into groups of BALB/c mice (10 per group) for 4 successive days beginning one day after tumour inoculation. ▽ Whole organism (OK-432), 100 µg/mouse. ○ LTA, 10 µg. □ Peptidoglycan, 10 µg. ◇ C-carbohydrate, 10 µg. △ M protein, 10 µg. ● Non-treated control. Significantly different from the control on day 60: $^{**}P<0.01$.

**Figure 2** Antitumour effects of streptococcal cellular components on solid Meth A. The schedule of administration of test materials was the same as that in **Figure 1,** except 8 mice were used in each group. ▽ Whole organisms, 100 µg. ○ LTA, 10 µg. □ Peptidoglycan, 10 µg. ◇ C-carbohydrate, 10 µg. △ M protein, 10 µg. ● Non-treated control. Significantly different from the control on day 34: $^{**}P<0.01$, $^{*}P<0.05$.

The antitumour effect of LTA as a function of dose was then studied. When 10 µg LTA were injected into mice inoculated with Meth A ascites (Figure 3), 4 out of the 10 mice survived >60 days without tumour. On the 60th day, the mean survival in the LTA-treated mice was 2.5 times longer than that of the non-treated control mice. Administration of either 50 µg LTA definitely prolonged the survival of tumour-inoculated mice. However, the antitumour effects in this group were less marked than those receiving 10 µg LTA.

With the solid Meth A, 10 µg LTA greatly inhibited tumour growth (77.3%) (Figure 4). Four out of 8 mice were tumour-free when evaluated 34 days after the inoculation. Administration of 2 µg LTA also significantly suppressed tumour growth (38.0%), but the extent of suppression was less than that caused by 10 µg LTA. In the case of 50 µg LTA, no antitumour effect was evident against solid Meth A.

In the experiment described above, the mice which were completely cured (tumour-free) after administration of LTA

**Figure 3** Antitumour effects of streptococcal LTA on Meth A ascites. See **Figure 1** for details of the LTA administration schedule (10 mice per group). ▽ 50 μg, □ 10 μg, △ 2 μg. ● Non-treated control. Significantly different from the control on day 60: $^{**}P<0.01$.

**Figure 4** Antitumour effects of streptococcal LTA on solid Meth A. See **Figure 1** for details of the LTA administration schedule (8 mice per group). ▽ 50 μg, □ 10 μg, △ 2 μg. ● Non-treated control. Significantly different from the control on day 34: $^{**}P<0.001$, $^{*}P<0.05$.

rejected rechallenge with Meth A. Moreover, none of the mice receiving test doses of LTA, showed any side effects such as diarrhoea, ataxia or anorexia.

### *Effect of LTA or other streptococcal components on Meth A tumour cells* in vitro

A possible direct cytotoxic effect of test streptococcal cellular components, especially LTA, was checked against Meth A tumour cells *in vitro*.

None of test streptococcal components exhibited any direct cytotoxic effects on Meth A tumour cells at any test dose levels (Table I).

### *TNF-inducing ability of streptococcal components*

The assay of the ability of test streptococcal components to induce TNF in the serum of the *P. acnes*-primed mice showed that only LTA among the test materials caused a high level of TNF activity. The extent of TNF induction by 100 μg LTA was comparable to that induced by 10 μg of a reference LPA (Table II).

**Table I** No direct cytotoxicity of streptococcal cellular components on Meth A cells *in vitro*

| Component | Dose ($\mu g\,ml^{-1}$) | $^3$H-TdR uptake[a] (mean ± s.d., cpm) | $^3$H-TdR uptake[a] Percent inhibition[b] |
|---|---|---|---|
| LTA | 100 | 204,590 ± 6,198 | −5.1 |
| | 2 | 196,025 ± 3,501 | −0.7 |
| | 0.04 | 188,213 ± 6,169 | −3.4 |
| C-carbohydrate | 100 | 196,140 ± 1,302 | −0.7 |
| | 2 | 202,331 ± 3,332 | −3.9 |
| | 0.04 | 205,899 ± 2,330 | −5.7 |
| Peptidoglycan | 100 | 176,027 ± 3,696 | 9.6 |
| | 2 | 199,367 ± 2,538 | −2.4 |
| | 0.04 | 195,440 ± 5,065 | −0.4 |
| M protein | 100 | 207,093 ± 212 | −6.4 |
| | 2 | 201,706 ± 3,416 | −3.6 |
| | 0.04 | 195,225 ± 4,419 | −0.3 |
| None | | 187,262 ± 3,501 | — |

Various dose levels of test materials were added to Meth A cells ($5\times10^3$ cells $ml^{-1}$ in RPMI 1640 medium supplemented with 8% FCS), and incubated at 37°C for 48 h in 5% $CO_2$-air. Then $^3$H-TdR (0.5 μCi/well) was added to those reaction mixtures. After a further 6 h of incubation, the incorporation of $^3$H-TdR into Meth A cells was measured of a liquid scintillation spectrometer. [a]In triplicate. [b](1 − cpm in test/cpm in 8% FCS-RPMI 1640 control) × 100 (%).

**Table II** The TNF-inducing ability of various streptococcal cellular components

| Component | Dose (μg/mouse) | $^3$H-TdR uptake inhibition, Serum dilution 1/100 | 1/500 | 1/2,500 | 1/12,500 |
|---|---|---|---|---|---|
| LTA | 100 | 97***[a] | 96** | 86** | 67* |
| Peptidoglycan | 100 | 5 | 6 | −5 | −4 |
| C-carbohydrate | 100 | 10 | −6 | 3 | −3 |
| M protein | 100 | 6 | 9 | NT[b] | NT |
| PGP | 100 | −9 | 10 | −1 | −9 |
| Nucleic acids | 100 | 18 | −15 | −23 | 10 |
| LPS (*S. enteritidis*) | 10 | 91** | 88** | 84** | 77* |

[a]Significantly different from the control: $^{**}P<0.001$, $^{*}P<0.01$. [b]Not tested.

### Discussion

Pieringen and Ganfield (1975) reported that LTA was anchored to bacterial cells by intercalation of the lipid end of the molecule into the phospholipid bilayer of the cytoplasmic membrane, with the linear PGP portion of the molecule penetrating through the cell wall peptidoglycan and protruding from the outermost cell surface. The present study revealed that LTA definitely inhibited the growth of both solid Meth A and Meth A ascites in BALB/c mice, just as whole organisms did. In a separate study, we found that PGP prepared by alkali treatment of LTA inhibited the growth of solid Meth A tumour, but not that of the ascites variant. Kigoshi (1971) reported that a lipid preparation extracted from *S. pyogenes* had antitumour effects on ascites-type Ehrlich carcinoma. It was also shown that unlike whole organisms, the cell wall had antitumour effects only on solid tumours and the cytoplasmic membrane had antitumour effects only on tumour ascites (Yamamoto *et al.* 1980). These findings may be explained by assuming that neither cell wall nor cytoplasmic membrane has intact LTA molecules.

The problem is to know the mechanism by which LTA causes tumour suppression. Direct killing of cells by LTA was excluded in the present study (Table I). In a previous study (Yamamoto *et al.*, 1985*a*), we demonstrated that the LTA of *S. pyogenes* was capable of inducing TNF in the sera of mice primed with *P. acnes,* and that the serum specimens induce haemorrhagic necrosis of pre-established Meth A tumour. Since the cytotoxic factor (CF) induced by LTA was almost completely neutralized with anti-TNF serum, we believe CF induced by LTA is different from lymphotoxin (to be published). The present study reconfirmed the above findings and showed further that several streptococcal components other than LTA were inactive in TNF induction (Table II). In conformity with this finding, these streptococcal components, unlike LTA, had no antitumour effects *in vivo*.

Activated cytotoxic macrophages are likely to play an important role in host defence against neoplasia and infections. Hamada *et al.* (1985) demonstrated that LTA induced the tumoricidal macrophages *in vitro*. Several research groups demonstrated that a soluble cytotoxic factor produced from activated macrophages is similar, if not identical, to tumour necrosis factor (Yamamoto *et al.*, 1986; Dryslade *et al.*, 1987; Hashimoto *et al.*, 1987).

The results obtained in this study indicate that LTA is one of the antitumour components of the streptococcal preparation, OK-432, and that one of the mechanisms of the antitumour effect of OK-432 may be the induction of TNF.

### References

DRYSDALE, B.-E., YAPUNDICH, R.A., SHIN, M.L. & SHIN, H.S. (1987). Lipopolysaccharide-mediated macrophage activation: The role of calcium in the generation of tumoricidal activity. *J. Immunol.*, **138**, 951.

FULLER, A.T. (1938). The formamide method for the extraction of polysaccharides from hemolytic streptococci. *Br. J. Exp. Path.*, **19**, 130.

HAMADA, H. & TORII, M. (1978). Effect of sucrose in culture media on the location of glucosyltransferase of *Streptococcus mutans* and cell adherences to glass surface. *Infect. Immuni.*, **20**, 592.

HAMADA, S., YAMAMOTO, T., KOGA, T., McGHEE, J.R., MICHALEK, S.H. & YAMAMOTO, S. (1985). Chemical properties and immunological activities of Streptococcal lipoteichoic acids. *Zbl. Bakt. Hyg. A.*, **123**, 1.

HASHIMOTO, S., NOMOTO, K., NAGAOKA, M. & YOKOKURA, T. (1987). *In vitro* and *in vivo* release of cytostatic factors from *Lactobacillis casei*-elicited peritoneal macrophages after stimulation with tumour cells and immunostimulants. *Cancer Immunol. Immunother.*, **24**, 1.

KIGOSHI, S. (1971). *In vitro* anti-tumor activity of lipid extracts from a group A streptococcus against Ehrlich ascites carcinoma in mice. *Experientia,* **27/8**, 976.

KOSHIMURA, S., RYOYAMA, K., OGAWA, H. & 4 others (1977). Antitumor activity of protoplast membrane from group A streptococcus. *Jpn. J. Exp. Med.*, **47**, 341.

KUROKAWA, T., HATTORI, T. & FURUE, H. (1972). Clinical experience with streptococcal anticancer preparation, OK-432 (BSC-B116209). *Cancer Chemother. Rep.*, **47**, 341.

MOSKOWITZ, M. (1966). Separation and properties of a red cell sensitizing substance from streptococci. *J. Bacteriol.*, **91**, 2200.

OFEK, I., BEACHEY, E.H., JEFFERSON, W. & CAMPBELL, G.L. (1975). Cell membrane-binding properties of group A streptococcal lipoteichoic acid *J. Exp. Med.*, **141**, 990.

PIERINGER, R.A. & GANFIELD, M.C.W. (1975). Phophatidylkojibiosyl diglyceride: Metabolism and function as an anchor in bacterial cell membrane. *Lipids,* **10**, 421.

SAKURAI, Y., TUKAGOSHI, S. & SAITO, S. (1972). Tumor-inhibitory effect of a streptococcal preparation (NSC-B116209). *Cancer Chemother. Rep.*, **256**, 9.

SCHLEIFER, K.H. & KANDLER, O. (1967). Zur chemichen zusammensetzung der zellwand der streptokokken. I. Die aminosaure sequentz des mureins von *Str. themophilus* und *Str. faecalis. Arch. Mikrobiol.*, **57**, 335.

USAMI, H. (1985). Biological and immunological properties of native Streptococcal M protein isolated by use of endo-N-acetylmuramidase, especially production of opsonic antibody. *Jpn. J. Bacteriol.*, **40**, 477.

WICKEN, A.J. & KNOX, K.W. (1980). Bacterial cell surface amphilies. *Biochem. Biophys. Acta,* **604**, 1.

YAMAMOTO, A., HOMMA, J.Y., USAMI, H. & SUGAWARA, Y. (1980). Production L-forms of *Streptococcus pyogenes* and their antitumor effect. *Jpn. J. Exp. Med.*, **50**, 383.

YAMAMOTO, A., USAMI, H., NAGAMUTA, M. & 6 others (1985a). The use of lipoteichoic acid (LTA) from 11 *streptococcus pyogenes* to induce a serum factor causing tumor necrosis. *Br. J. Cancer,* **51**, 739.

YAMAMOTO, A., NAGAMUTA, M., USAMI, H. & 4 others (1985b). Production of cytotoxic factor into mouse peritoneal fluid by OK-432, a streptococcal preparation. *Immunol. Lett.*, **11**, 83.

YAMAMOTO, A., NAGAMUTA, M. USAMI, H. & 4 others (1986). Release of tumor necrosis factor (TNF) into mouse peritoneal fluids by OK-432, a streptococcal preparation. *Immunopharmacol.*, **11**, 79.